# The Practitioner’s Reference Book

**Published:** 1879-04

**Authors:** 


### BIBLIOGRAPHICAL.

---

The Practitioner's Reference Book. Adapted to the use of the Physician, the Pharmacist and the Student. By Richard J. Dunglison, M.D. Lindsay & Blakiston, Philadelphia. 1877.

This book of three hundred and forty-one pages is the most handy book for reference that we have met with. It contains: General Information for the Practitioner, Therapeutical and Practical Hints, Dietetic Rules and Precepts, How to Conduct a Post-Mortem Examination, with an Introduction consisting of the memorable Hippocratic oath.

Under these several headings exceedingly interesting and important details are discussed, rendering the work very practical and desirable.

The publishers, Messrs. Lindsay & Blakiston, have gotten it up in their usual happy style, thereby rendering the book both useful and ornamental.

